# Effects of ketosis in mitochondrial myopathy: potential benefits of a mitotoxic diet

**DOI:** 10.15252/emmm.201606933

**Published:** 2016-10-11

**Authors:** Robert DS Pitceathly, Carlo Viscomi

**Affiliations:** ^1^ MRC Centre for Neuromuscular DiseasesUCL Institute of Neurology and National Hospital for Neurology and NeurosurgeryLondonUK; ^2^ Department of Basic and Clinical NeuroscienceInstitute of Psychiatry, Psychology and NeuroscienceKing's College LondonLondonUK; ^3^ MRC‐Mitochondrial Biology UnitCambridgeUK

**Keywords:** Genetics, Gene Therapy & Genetic Disease, Metabolism

## Abstract

The field of mitochondrial medicine is rapidly transitioning from preclinical observation to clinical application. Translation of promising data obtained in mouse models is not always straight‐forward, however. Building on their own work showing that a ketogenic diet induces mitochondrial biogenesis and delays the onset of disease in the Deletor mouse, Ahola *et al* administered modified Atkins diet (mAD) to five patients with mitochondrial myopathy caused by mitochondrial DNA deletions (Ahola *et al*, 2016). Surprisingly, mAD did not induce mitochondrial biogenesis in patients, but rather triggered the progressive damage of muscle cells, particularly those with impaired respiratory chain activity (the ragged‐red fibres). The subsequent extensive characterisation of the metabolic and molecular profile changes observed in patients and healthy controls provides a significant advance towards understanding the feasibility of dietary modification as a treatment strategy for mitochondrial diseases.

Myopathy is a recurrent feature of mitochondrial disorders, a group of highly heterogeneous genetic syndromes directly or indirectly related to impaired respiratory chain function (Zeviani & Di Donato, 2004). Progressive external ophthalmoplegia (PEO) is the most common clinical manifestation of mitochondrial disease. Exercise intolerance and fatigue are commonly associated with PEO and frequently have a significant impact on the quality of life of patients (Chinnery, 2015). There are no approved effective therapies available for mitochondrial diseases and clinical management is largely supportive, although a number of vitamins and co‐factors are often prescribed (Viscomi *et al*, 2015). The majority of interventions proposed have been applied to genetically heterogeneous cohorts of patients with limited pre‐ and post‐treatment molecular and metabolic characterisation. In this issue of *EMBO Molecular Medicine*, Ahola and colleagues address this important issue by extensively detailing the pathological, metabolic and transcriptomic consequences of a ketogenic (high fat, low carbohydrate) modified Atkins diet (mAD) in both healthy subjects and a small cohort of patients with PEO and exercise intolerance caused by single or multiple mitochondrial DNA (mtDNA) deletions (Ahola *et al*, 2016).

Despite uncertainty surrounding the precise mechanism, ketogenic diet (KD) has been used since the 1920s to treat seizures in drug‐resistant epilepsy (Paoli *et al*, 2013), including Alpers–Huttenlocher syndrome, a severe encephalopathy in patients harbouring mutations in the mtDNA polymerase gamma, *POLG,* gene (Joshi *et al*, 2009). Preclinical evidence to support the beneficial effects of a KD in mitochondrial disease includes the discovery that ketogenic medium kills cybrid cell lines carrying 100% deleted mtDNA and reduces the amount of deleted mtDNA in heteroplasmic lines (Santra *et al*, 2004). Furthermore, KD has been proven to induce mitochondrial biogenesis and slow down disease progression in the Deletor mouse, which develops a gradually progressive myopathy due to mutant form of the mitochondrial helicase TWINKLE (Ahola‐Erkkila *et al*, 2010).

mAD induces ketosis, a metabolic state characterised by the production of ketone bodies from acetyl‐CoA. Acetyl‐CoA accumulation results from reduced availability of oxaloacetate, which is diverted towards hepatic gluconeogenesis when dietary glucose is restricted. Ketone bodies are released into the bloodstream and re‐uptaken by non‐gluconeogenic tissues, such as the brain, where oxaloacetate is available, and where they are subsequently re‐converted into acetyl‐CoA entering the Krebs cycle (Owen *et al*, 1967).

The five patients with mitochondrial disease presented by Ahola *et al* (2016) unexpectedly developed acute muscle damage with burning sensation and muscle pain spreading from their lower limbs to the back, with elevated creatine kinase (CK), transaminases and myoglobin levels within days from initiating the mAD regimen, unlike their age‐ and sex‐matched controls (Fig 1). Cessation of the mAD quickly reversed these symptoms. Further investigations revealed that mAD induced selective necrotic degeneration of ragged‐red fibres (RRF) in PEO patients, without significant changes in the background levels of apoptosis and autophagy or further activation of satellite cells. No toxic effect was observed in muscle fibres of healthy individuals.

**Figure 1:**
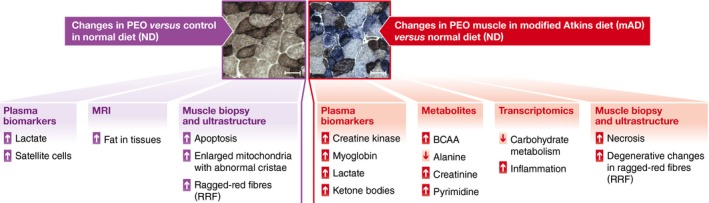
Main changes induced by mAD in myopathic patients

Perhaps even more surprisingly, at 2.5‐year follow‐up muscle strength had mildly improved in three of four patients. Unfortunately, muscle tissue was unavailable for further evaluation at this time point. Nevertheless, the analysis of the metabolic and molecular changes occurring during the acute phase in patients and controls is highly informative. First, as a result of impaired respiratory chain function, the metabolism of patients with PEO relied heavily on glucose, as indicated by their high lactate levels. In addition, lipids could not be utilised and therefore accumulated in the liver, visceral depots and skeletal muscle tissue. Introduction of the mAD regimen increased plasma β‐hydroxybutyrate levels similarly in both controls and patients, suggesting comparable ketosis induction in both groups. However, due to the patients' inability to catabolise ketone bodies, a metabolic crisis appeared to be triggered within their muscle tissue. Second, patients and controls responded with similar metabolic profiles to the switch from an isocaloric to a high‐fat, low‐carbohydrate diet. Increased plasma ammonium, a marker of amino acid consumption for energy production, and reduction in body weight and fat depots were similar in patients and controls. Third, transcriptomic analysis demonstrated mAD increased mitochondrial biogenesis, in addition to pathways related to lipid utilisation, in control muscle. Contrariwise, these pathways were blunted in the muscle tissue of patients with PEO whose mitochondria were unable to metabolise lipids, while pathways related to inflammation were activated in agreement with ongoing damage. Finally, metabolomic profiling revealed that patients and controls responded similarly to mAD by increasing the carnitine carrier pool and upregulating alternative pathways in order to generate energy, such as the branched chain amino acids. Notably, alanine, a biomarker of mitochondrial disease, and other disease‐associated metabolites, such as asparagine and xanthine, were normalised by mAD.

Interestingly, unlike the reported human study, a short‐term ketogenic diet in the Deletor mice revealed no signs of muscle degeneration or creatine kinase elevation, suggesting that mice with mitochondrial disease have the metabolic capacity to adapt more readily to dietary changes than humans.

This study significantly advances current knowledge concerning the molecular, cellular and systemic adaptations which occur in the tissues of PEO patients, emphasises the concept that any dietary intervention in mitochondrial disease necessitates careful clinical evaluation and laboratory monitoring, and reaffirms the caution required when extrapolating animal data to human studies.

Analysis of muscle tissue obtained after 2.5 years would have been extremely informative in further understanding the progression of mitochondrial disease after mAD and would have clarified a number of important questions which remain unanswered: Is there a clear and persistent reduction in RRF? If so what is the mechanism for this phenomenon? Does muscle regeneration occur via activation of muscle satellite cells? Although the latter seems unlikely given the data obtained during the acute phase of the study, it remains possible that over time satellite cells replace the necrotic muscle fibres, thus explaining the observed clinical amelioration. In support of this possibility is the reported evidence that satellite cells have lower levels of deleted mtDNA molecules compared to the fully differentiated myocytes and, once activated, can proliferate and fuse with existing muscle fibres replacing damaged cells (Moraes *et al*, 1989; Clark *et al*, 1997). In conclusion, this study implies that mAD potentially represents an effective strategy to initiate the selective death of RRF, with subsequent replacement of necrotic fibres by healthy muscle tissue generated from satellite cells.

Further work is warranted to determine whether repeated cycles of mAD could benefit patients via selective elimination of defective muscle fibres.

## References

[emmm201606933-bib-0001] Ahola S , Auranen M , Isohanni P , Niemisalo S , Urho N , Buzkova J , Velagapudi V , Lundbom N , Hakkarainen A , Muurinen T *et al* (2016) Modified Atkins diet induces subacute selective ragged‐red‐fiber lysis in mitochondrial myopathy patients. EMBO Mol Med 8: 1234–1247 27647878 10.15252/emmm.201606592 PMC5090657

[emmm201606933-bib-0002] Ahola‐Erkkila S , Carroll CJ , Peltola‐Mjosund K , Tulkki V , Mattila I , Seppanen‐Laakso T , Oresic M , Tyynismaa H , Suomalainen A (2010) Ketogenic diet slows down mitochondrial myopathy progression in mice. Hum Mol Genet 19: 1974–1984 20167576 10.1093/hmg/ddq076

[emmm201606933-bib-0003] Chinnery PF (2015) Mitochondrial disease in adults: what's old and what's new? EMBO Mol Med 7: 1503–1512 26612854 10.15252/emmm.201505079 PMC4693502

[emmm201606933-bib-0004] Clark KM , Bindoff LA , Lightowlers RN , Andrews RM , Griffiths PG , Johnson MA , Brierley EJ , Turnbull DM (1997) Reversal of a mitochondrial DNA defect in human skeletal muscle. Nat Genet 16: 222–224 9207784 10.1038/ng0797-222

[emmm201606933-bib-0005] Joshi CN , Greenberg CR , Mhanni AA , Salman MS (2009) Ketogenic diet in Alpers‐Huttenlocher syndrome. Pediatr Neurol 40: 314–316 19302948 10.1016/j.pediatrneurol.2008.10.023

[emmm201606933-bib-0006] Moraes CT , Schon EA , DiMauro S , Miranda AF (1989) Heteroplasmy of mitochondrial genomes in clonal cultures from patients with Kearns‐Sayre syndrome. Biochem Biophys Res Commun 160: 765–771 2541710 10.1016/0006-291x(89)92499-6

[emmm201606933-bib-0007] Owen OE , Morgan AP , Kemp HG , Sullivan JM , Herrera MG , Cahill GF Jr (1967) Brain metabolism during fasting. J Clin Invest 46: 1589–1595 6061736 10.1172/JCI105650 PMC292907

[emmm201606933-bib-0008] Paoli A , Rubini A , Volek JS , Grimaldi KA (2013) Beyond weight loss: a review of the therapeutic uses of very‐low‐carbohydrate (ketogenic) diets. Eur J Clin Nutr 67: 789–796 23801097 10.1038/ejcn.2013.116 PMC3826507

[emmm201606933-bib-0009] Santra S , Gilkerson RW , Davidson M , Schon EA (2004) Ketogenic treatment reduces deleted mitochondrial DNAs in cultured human cells. Ann Neurol 56: 662–669 15389892 10.1002/ana.20240

[emmm201606933-bib-0010] Viscomi C , Bottani E , Zeviani M (2015) Emerging concepts in the therapy of mitochondrial disease. Biochim Biophys Acta 1847: 544–557 25766847 10.1016/j.bbabio.2015.03.001

[emmm201606933-bib-0011] Zeviani M , Di Donato S (2004) Mitochondrial disorders. Brain 127: 2153–2172 15358637 10.1093/brain/awh259

